# Cascading from SARS-CoV-2 to Parkinson’s Disease through Protein-Protein Interactions

**DOI:** 10.3390/v13050897

**Published:** 2021-05-12

**Authors:** Ernesto Estrada

**Affiliations:** 1 Institute of Mathematics and Applications, University of Zaragoza, Pedro Cerbuna 12, 50009 Zaragoza, Spain; estrada66@unizar.es; 2 ARAID Foundation, Government of Aragon, 50018 Zaragoza, Spain; 3 Institute for Cross-Disciplinary Physics and Complex Systems (IFISC, UIB-CSIC), Campus Universitat de les Illes Balears, E-07122 Palma de Mallorca, Spain

**Keywords:** COVID-19, Parkinson’s disease, protein–protein interactions, exosomes, molecular mechanisms, post-translational modifications

## Abstract

Extensive extrapulmonary damages in a dozen of organs/systems, including the central nervous system (CNS), are reported in patients of the coronavirus disease 2019 (COVID-19). Three cases of Parkinson’s disease (PD) have been reported as a direct consequence of COVID-19. In spite of the scarce data for establishing a definitive link between COVID-19 and PD, some hypotheses have been proposed to explain the cases reported. They, however, do not fit well with the clinical findings reported for COVID-19 patients, in general, and for the PD cases reported, in particular. Given the importance of this potential connection, we present here a molecular-level mechanistic hypothesis that explains well these findings and will serve to explore the potential CNS damage in COVID-19 patients. The model explaining the cascade effects from COVID-19 to CNS is developed by using bioinformatic tools. It includes the post-translational modification of host proteins in the lungs by viral proteins, the transport of modified host proteins via exosomes out the lungs, and the disruption of protein-protein interaction in the CNS by these modified host proteins. Our hypothesis is supported by finding 44 proteins significantly expressed in the CNS which are associated with PD and whose interactions can be perturbed by 24 host proteins significantly expressed in the lungs. These 24 perturbators are found to interact with viral proteins and to form part of the cargoes of exosomes in human tissues. The joint set of perturbators and PD-vulnerable proteins form a tightly connected network with significantly more connections than expected by selecting a random cluster of proteins of similar size from the human proteome. The molecular-level mechanistic hypothesis presented here provides several routes for the cascading of effects from the lungs of COVID-19 patients to PD. In particular, the disruption of autophagy/ubiquitination processes appears as an important mechanism that triggers the generation of large amounts of exosomes containing perturbators in their cargo, which would insult several PD-vulnerable proteins, potentially triggering Parkinsonism in COVID-19 patients.

## 1. Introduction

The coronavirus disease 2019 (COVID-19) has become a global pandemic with an estimated 80 million confirmed cases and 1.76 million deaths (as of 28 December 2020). The disease, which is produced by the Severe Acute Respiratory Syndrome coronavirus-2 (SARS-CoV-2) [1,2,3,4], is characterized by affecting a dozen of extrapulmonary organs/systems in several patients [5,6,7], with residual symptoms manifested even after the virus is not detectable (see, for instance, References [8,9]). Particularly, neurologic complications have emerged as an increasingly recognized cause of morbidity and mortality in COVID-19 patients [5,10,11,12]. The most common of these neurologic symptoms include cerebrovascular events, encephalitis, Guillain-Barre syndrome, acute necrotizing encephalopathy, hemophagocytic lymphohistiocytosis, and acute ischemic cerebrovascular syndrome, as well as neuropsychiatric symptoms, such as dizziness, disturbed sleep, cognitive deficits, delirium, hallucinations, and depression [5,10,11,12]. It has been stressed that these damages may substantially increase the incidence of neurodegenerative diseases and promote dementia [13].

Recently, three independent case reports have described the development of Parkinson’s disease (PD) following COVID-19 [14,15,16]. The cases refer to a 35-year-old female [14] and two men of 45 [15] and 58 years old [16], respectively. The three patients were previously healthy and had no family history of Parkinsonism. Some remarkable common characteristics of the three case reports: (i) normal cerebrospinal fluid (CSF) analysis, and (ii) no relevant findings in magnetic resonance imaging (MRI). For instance, although anti-SARS-CoV-2 IgG were detected in serum of the 45-year-old patient, it was not detected in CSF, which was also negative for SARS-CoV-2 in real-time RT-PCR. PCR assay of CSF for SARS-CoV-2, as well as other tests for several microbiological organisms, which were also negative. An intriguing finding was reported by Méndez-Guerrero et al. [16] in the analysis of the CSF where they found no explanation for the high protein levels in this fluid in spite of the fact that neither the blood-brain barrier dysfunctions nor oligoclonal bands were detected in the CSF. Additionally, Cohen et al. [15] reported for their patient negative evidences for mutations in common hotspots for PD, as well as in Next Generation Sequencing tests for genes related to this disease. There have been debate about the causal-relation between COVID-19 and these three cases of Parkinsonism [17,18,19,20,21]. In the meantime, a case of acute Parkinsonism was reported by Akilli and Yosunkaya [22].

In their response to criticisms, Méndez-Guerrero et al. replied that they “only described an intriguing case that might raise interesting questions from the scientific community” [19]. In this direction, three main hypotheses have been formulated to explain the development of post-COVID-19 PD [20]. The first is based on vascular insults on the brain, which could directly damage the nigrostriatal system. The second appeals to systemic inflammation possible due to cytokine storm causing neuroinflammation and death of nigral dopamine neurons. The third claims that SARS-CoV-2 is a neurotropic virus entering and damaging directly the brain. The three hypothesis are supported by some clinical facts, but rejected by some others. For instance, autopsy studies have not reported any bleeding or small thrombosis in the brain, which point out against the first hypothesis [23]. Recent meta analysis in a large number of patients has strongly questioned the presence of cytokyne storm in patients with severe COVID-19 [24,25,26,27,28], which makes it difficult to support the second hypothesis. Finally, the three previously mentioned reports of post-COVID-19 PD [14,15,16] did not find SARS-CoV-2 in CSF, although it was present in blood. It has been stressed by Merello et al. [29] that the evidences of these three cases is too limited to link COVID-19 and PD. However, we provide here strong molecular evidence that such a link could exist through cascading effects taking place through the protein-protein interaction (PPI) networks that interconnect SARS-CoV-2 perturbations in the lungs with potential extrapulmonary damages.

## 2. Antecedents

In proposing the new hypothesis detailed in this work, we are based on a series of antecedents which provide support to the necessity of developing a novel hypothesis that explains extrapulmonary damages in COVID-19 patients, as well as to the individual elements conforming the current hypothesis. Here, we provide a resume of these antecedents.

- Several viruses have been associated with Parkinsonism [30], which include RNA viruses of the families of Bornaviridae, Orthomyxoviridae, Paramyxoviridae, Picornavirisae, Retroviridae, and Flaviviridae. After the 1918 pandemic influenza outbreak caused by H1N1 influenza virus, there were several cases of postencephalic Parkinsonism [31]. Recently, Jang et al. [32] have reported that a highly pathogenic H5N1 influenza virus can induce Parkisonian pathology in mice.
- The cytokine storm hypothesis is not able to explain the extrapulmonary damages produced by SARS-CoV-2 infection as the median levels of IL-6 in patients with severe COVID-19 are 10- to 200-fold smaller (see Table 1) than those observed in patients with hyperinflammatory phenotype of acute respiratory distress syndrome (ARDS) (see also the comments in References [33,34]):

**Table 1:** Plasma level of Interleukin-6 (IL-6) reported in severe cases of COVID-19, as well as in “hypo” and hyperinflammatory processes in acute respiratory distress syndrome (ARDS). * Critically ill cases.

Severe COVID-19	Population		IL-6 Level pg/mL	
[35]	84		7 (6–11)	
[36]	54		11 (8–14)	
[37]	286		25 (10–55)	
[38]	237		26 (11–69)	
[39]	85		64 (31–165)	
[40]	17 *		64 (25.6–111.9)	
ARDS	“hypoinflamatory”		hyperinflamatory	
	pop.	IL-6 level pg/mL	pop.	IL-6 level pg/mL
[41]	638	86 (34–216)	246	578 (181–2621)
[42]	386	154 (67–344)	135	1525 (584–3802)
[43]	451	282 (111–600)	269	1618 (517–3205)

- SARS-CoV-2 vRNA has been detected using PCR techniques in different parts of the brain [44,45]. While the number of copies per mL of RNA from homogenized organs and tissues in 11 patients deceased from COVID-19 ranged 114.8–19,498 for different parts of the respiratory system, it ranged only 2.3–4.9 for different parts of the nervous system [44].
- It has been recently determined by Philippen et al. [46] that SARS-CoV-2 infection causes brain inflammation in the macaque model. Post-mortem analysis demonstrated infiltration of T-cells and activated microglia in the brain. Viral RNA was detected in brain tissues from one animal. The authors observed Lewy bodies in brains of all rhesus macaques. In humans, Lewy body formation is an indication for the development of Parkinson’s disease.
- Wölfel et al. [47] reported infectious virus readily isolated from samples derived from the throat or lung of COVID-19 patients, but not from stool samples—in spite of high concentrations of virus RNA. Blood and urine samples never yielded virus. Therefore, the identification of vRNA does not necessarily indicates viral tropism. There is huge evidence that RNA, including viral one, can be delivered to mammalian cells by means of extracellular vesicles, such as exosomes [48].
- It is known today that exosomes [49] facilitate the spread of viruses improving virus infection pathogenesis [50,51,52]. For instance, proteins and noncoding RNA from HIV are known to be transported by exosomes to (i) increase susceptibility to infection, (ii) influence virus budding and spread, and (iii) increase neuropathogenesis. Viral RNA and proteins from Zika virus are transported by exosomes to improve viral spread to neighboring cells. Virus spread is also known to be facilitated by exosomes in EV-A71, Rabies virus, and HCV. The use of exosomes containing viral proteins and/or viral RNA is also know to evade the immune system in the case of EBV, KSHV, HSV1, HTLV-1, and avian influenza (H5N1).
- Ramakrishnaiah et al. [53] found experimentally that purified exosomes isolated from HCV-infected human cells contained full-length viral RNA and proteins, which were capable of transmitting infection to human cells. They also shown that exosomes-transmitted infection was resistant to antibody neutralization.
- The potential role of exosomes and extracellular vesicles in COVID-19 has been previusly proposed by several authors [54,55,56].
- V’kovski et al. [57] have shown recently that SARS-CoV-2 replicated to higher titers when infections were performed at 33 °C rather than 37 °C. The reverse is found for SARS-CoV. They also found that SARS-CoV-2 triggered a pronounced antiviral and pro-inflammatory response earlier and more strongly induced at 37 °C than at 33 °C. These temperatures correspond to the ones of the upper (33 °C) and lower (37 °C) respiratory tract.
- The temperature of healthy brain is slightly higher than 37 °C (ventral striatum 37.6 °C; dorsal striatum 37.2 °C; cerebellum 37.3 °C) [58], (frontal lobe (37.2 ± 0.6 °C) and thalamus (37.7 ± 0.6 °C)) [59], which may indicate a limited capacity for SARS-CoV-2 for reproducing in this organ where it also would trigger higher immunological response.
- Post-translational modification of host proteins is a key strategy of viral pathogens to modulate host factors critical for infection, which are essential for viruses’ replication, propagation, and evasion from host immune responses [60].
- It is today understood that the spread of perturbations across the subcellular networks, such as protein-protein interaction networks, is one of the major causes of diseases [61,62]. Such perturbations can be either of topological nature, e.g., deletion of nodes (proteins) or edges (interactions), or dynamical, i.e., the propagation of changes in the concentrations of given proteins in the cell.

## 3. Materials and Methods

### 3.1. Cascading Mechanism

**Figure 1:**
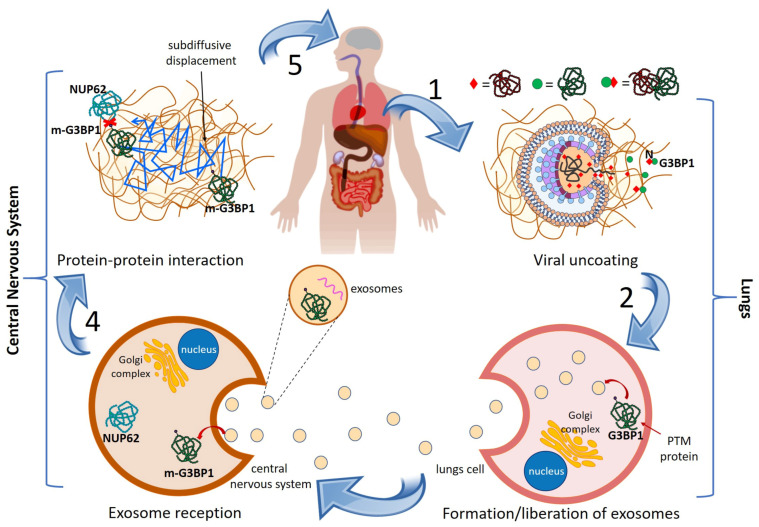
General mechanism of lungs-to-CNS cascade giving rise to PD in COVID-19 patients. See text for explanation.

We start by formulating our general mechanistic hypothesis to explain the propagation of damages from the lungs of COVID-19 patients to their central nervous system (CNS) and potentially produce PD. The pillars of this hypothesis are (see more details in the previous Section and in References [54,63]): (1) the existence of scientific evidence indicating that the disruption of PPIs is a major cause of diseases [62,64]; (2) the identification of human proteins targeted by SARS-CoV-2 proteins [65]; (3) the existence of evidence that SARS-CoV-2 proteins produce post-translational modification (PTM) of some human proteins, which may modify their capacity to interact with other proteins [66]; (4) the existence of cross-organ-talk mechanisms based on exosomes allowing the inter-organs transmission of effects [67]; and (5) the identification of a large number of genes involved in PD [68]. To simplify, we will describe the general mechanism explaining post-COVID-19 PD from one example and then generalize it. After the entrance of SARS-CoV-2 in the human body, it discharges its RNA and proteins in the lungs, as illustrated in Figure 1 (step 1). One of these viral proteins is the nucleocapside protein N, which is known to target, among others, the human protein G3BP1. As a result of this N-G3BP1 interaction, the spatial, physical, and/or chemical properties of G3BP1 are altered. These types of post-translational modifications (PTM) include structural modifications, such as phosphorylation, acetylation, acylation, glycosidation, ubiquitination, deamination, disulfide bond formation, and several others [69]. They may give rise to protein alterations that include up- or down-regulation, misfolding, alteration of active site or other critical regions, incorrect localization, and incorrect assembling, which can get involved in the development of diverse diseases [70]. Let us designate the PTM protein by m-G3BP1. The m-G3BP1 protein is known to be encapsulated in exosomes, as illustrated in Figure 1 (step 2). Exosomes are membrane-bound spherical extracellular vesicles of endocytic origin having diameters between 40 and 120 nm [71]. They contain cargoes of micro-RNA, proteins and lipids which can be transported unaltered at long distances in the body allowing inter-organ cross-talk. Exosomes are abundant in circulation, with estimates of 3 million exosomes per microliter of blood serum [71]. Today, it is widely recognized that exosomes play a fundamental role in neurodegenerative disorders, such as PD [71,72,73]. For instance, it has been shown that exosomes from CSF of PD patients are able to induce oligomerization of α-synuclein (α-syn) when compared with control CSF [74]. More recently, Han et al. [73] demonstrated that intravenous or intrastriatal injection of exosomes from PD patient serum in mice evokes protein aggregation, trigger dopamine neuron degeneration, induce microglial activation, and cause apomorphine-coaxed rotation and movement defects. The presence of “do not eat me” signals [75], i.e., CD47, on exosomal membranes protects them from phagocytes and improves their stability in circulation. This may be a plausible cause why Mendez-Guerrero et al. found high protein levels in the CSF without any dysfunction of the blood-brain barrier. Then, we suggest that m-G3BP1 can be delivered unaltered to the CNS by exosomes as illustrated in Figure 1 (step 3). G3BP1 is known to interact with several other proteins, including NUP62, which is highly expressed in the brain. However, due to the PTM of G3BP1, the interaction of m-G3BP1 with NUP62 is disrupted (see Figure 1, step 4), which is equivalent to remove the corresponding protein-protein interaction (PPI) from the human PPI network [76]. It has been reported that modifications in NUP62 are involved in PD (see Figure 1, step 5), completing a cascade from viral infection in the lungs to PD. We will detail in Discussion the mechanism involving G3BP1 and NUP62 in PD and provide the appropriate references therein.

### 3.2. Identification of VP and Their Perturbators

We start by considering the set $P_{1}$ of all 332 human proteins that directly interact with SARS-CoV-2 proteins found by Gordon et al. [65]. We then find the set $P_{2}\subseteq P_{1}$ of proteins targeted by viral proteins which are significantly expressed in the lungs. This is carried out by interrogating each protein in $S_{1}$ in the database The Human Protein Atlas [77] (https://www.proteinatlas.org/ accessed on 15 December 2020). A protein was considered as significantly expressed in the lungs if its RNA expression reported in Genotype-Tissue Expression (GTEx) has pTPM larger than 10, where pTPM is the protein-coding transcripts per million. A previous report uses pTPM larger than 5 [78], so we were much more restrictive here.

The set $P_{3}\subseteq P_{2}\subseteq P_{1}$ is found by interrogating each of the proteins in $P_{2}$ in the database Exocarta [79] (http://www.exocarta.org/ accessed on 15 December 2020). Any experimental report of the transport of the corresponding protein via exosomes in humans was considered as a positive evidence. Therefore, the proteins in $P_{3}$ are those which: (i) interact directly with SARS-CoV-2 proteins, (ii) are significantly expressed in the lungs and (iii) are found to form exosomes in humans. Each protein in $P_{3}$ was interrogated by the STRING database [80] (https://string-db.org/ accessed on 15 December 2020) to find all human proteins that interact with them. The new set $V_{1}$ is formed by 278 proteins.

Every protein in $V_{1}$ was interrogated with version 7.0 of DisGeNET [81], which contains 1,134,942 gene-disease associations (GDAs), between 21,671 genes and 30,170 diseases, disorders, traits, and clinical or abnormal human phenotypes, and 369,554 variant-disease associations (VDAs), between 194,515 variants and 14,155 diseases, traits, and phenotypes (https://www.disgenet.org/home/ accessed on 15 December 2020). We only interrogate these proteins for “Parkinson disease”, although other “Parkinson”-related entries exists in the database. All proteins in $S_{1}$ which were reported as associated with PD in DisGeNet form the set $S_{2}\subseteq S_{1}$. It contained 59 proteins which were potential candidates to be PD-VP. We then curate by hand each of these proteins and verify in the papers provided by DisGeNet whether there was strong evidence for considering them to be associated with PD. In this search we found 15 proteins for which DisGeNet contains errors that exclude them to be PD-VP. These proteins are: RING, PRKAR1A, PRKAR2A, RUNX1T1, RAB6B, NXF1, RAN, VPS11, NDUF57, SART3, RAB6A, RAB1B, RTN4R, SLC2A4, and USO1. The remaining 44 proteins form the set $S_{3}\subseteq S_{2}\subseteq S_{1}$ which are considered here as PD-vulnerable proteins (VPs). The set of perturbators $P_{4}\subseteq P_{3}$ is then formed by all proteins in $P_{3}$ which interact directly with a protein in $S_{3}$.

## 4. Results

According to the mechanism explained in Methods, after viral uncoating and liberation of viral proteins, some human proteins significantly expressed in the lungs are modified by their interactions with viral ones. Some of these PTM human proteins will travel to the CNS using exosomes and taking advantage of the increased permeability of the lungs caused by the viral infection [82]. Due to their PTM, some PPI in the CNS will be disrupted, including some of relevance for PD. Based on the previous mechanism, we conducted an intensive bioinformatic search consisting of the following steps. First, we identified all human proteins which fulfill the following requirements: (i) being directly targeted by a SARS-CoV-2 protein; (ii) being significantly expressed in the lungs; (iii) being found in exosomes in human tissues; (iv) being reported to form a PPI complex with at least one protein reported to have significant association with the development of PD. We call the proteins fulfilling these requirements “perturbators”. In total, we identified 24 perturbators which fulfill these requirements. In Table 2, we list these 24 proteins and their biological functions according to Gordon et al. [65], together with the protein and RNA expressions of each of them in the lungs. These 24 host proteins interact directly with 16 SARS-CoV-2 proteins as determined experimentally by Gordon et al. [65].

**Figure 2:**
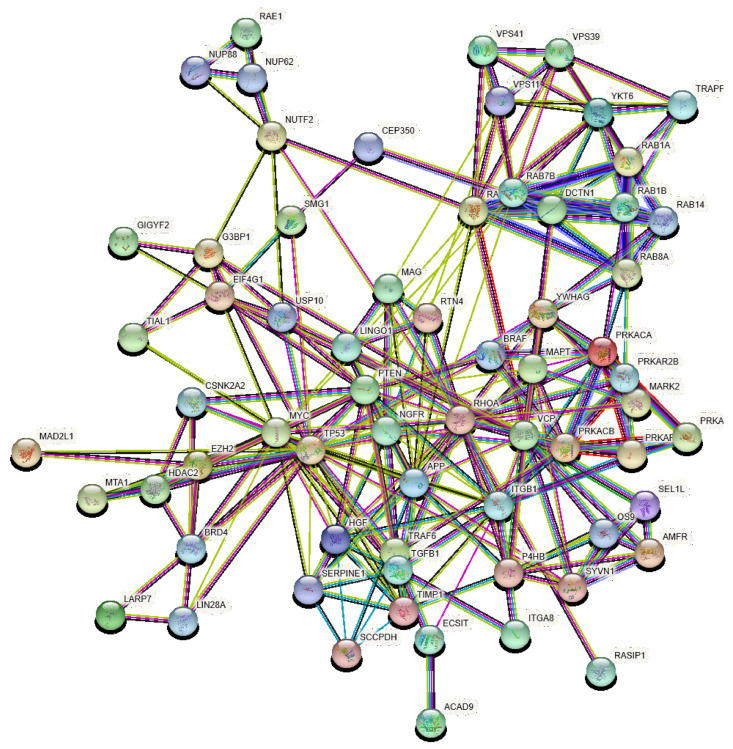
Protein-protein interaction network of all PD-VPs identified in this work and their perturbators. The network is built using STRING, and the edges are colored according to the way in which the corresponding PPI was determined: cyan, from curated databases; magenta, experimentally determined; green, gene neighborhood; red, gene fusions; blue, gene co-occurrence; lemon green, gene neighborhood; black, gene fusions; violet, gene co-occurrence.

Using the approach described in Methods, we identified 44 PD-VPs. These proteins, which are all associated with PD, are perturbed by the 24 host perturbators found here. There are two proteins, PRKACA and PRKAR2B, which are significantly expressed in the lungs, as well as in the CNS. They have been identified here as both perturbators and PD-VP. Thus, in general, we use the markers “_L” and “_CSN” to distinguish the location of the proteins. Considering the PPIs of the non-repeated 66 proteins, we obtain a network which includes the PD-VP and their perturbators, as illustrated in Figure 2. This network has 258 edges representing PPIs between of type P-P, P-VP, and VP-VP, where P: perturbator and VP: vulnerable protein. If we consider human PPI [76] and select a random cluster of proteins of similar size, the expected number of edges obtained should be equal to 125. This difference is statistically significant with *p*-value of $10^{-16}$ (see https://string-db.org/ for details, accessed on 15 December 2020). This means that the cluster of perturbators and VP have more interactions among themselves than what would be expected for a random set of proteins of similar size, drawn from the genome. This enrichment indicates that the proteins in this cluster are at least partially biologically connected, as a group.

**Figure 3:**
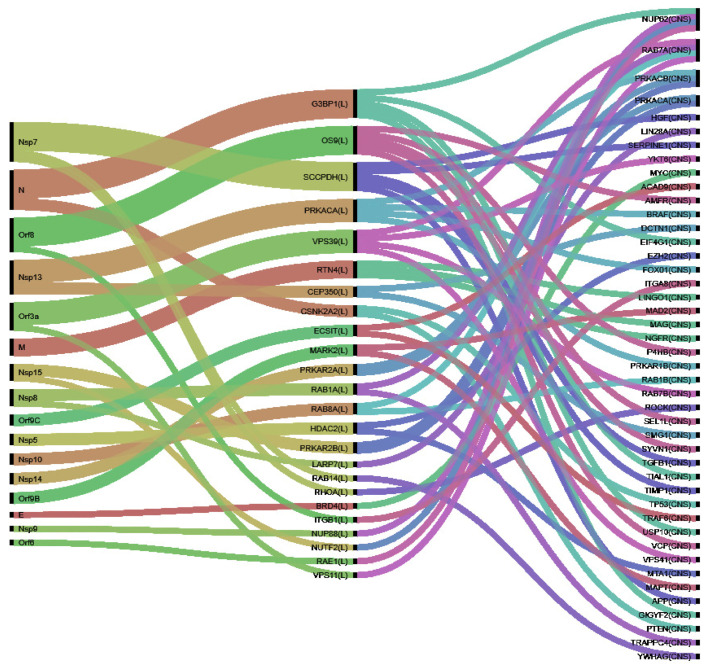
Cascading effects from SARS-CoV-2 proteins (**left**) to perturbators (**middle**) and from them to PD-VPs (**right**).

In Figure 3, we illustrate the connections between the three classes of proteins: viral proteins which modify the perturbators, which finally alter VPs by means of disruption of PPIs. We will further discuss in the next section the mechanisms implicating these VPs in PD and how their perturbators play a role in the disease development.

## 5. Discussion

Among the VPs, Rab7A and NUP62 (p62) are the ones having the largest number of potentially perturbed interactions. These proteins interact with four perturbators each. In the case of Rab7A, the perturbators are Rab1A, Rab8A, Vps11, and Vps39. Rab7A is a relatively small protein (208 amino acids) which is mainly found in late endosomes and which has been recognized as the only lysosomal Rab protein in the Rab GTPase superfamily [83]. It increases the degradation of α-syn aggregates whose presence in the brain is one of the main features of PD [83,84]. Rab7A reduces the proportion of cells with α-syn particles, as well as the amount and toxicity of α-syn. Therefore, the overexpression of Rab7A has been found beneficial on PD [83,84]. The mechanism by which Rab7A drops α-syn in cells is mediated by autophagy, and requires the autophagosome maturation, which consists on the fusion of autophagosomes and lysosomes to form autolysosomes. The last step allows autophogosomes to obtain hydrolytic enzymes indispensable for subsequent autophagic degradation. In fact, Rab7A is involved in governing several cellular processes, such as early-to-late endosome transition, biogenesis of lysosomes, transport of autophagosomes to endosomes/lysosomes, and vacuole formation. A crucial step in the early-to-late endosome maturation is the Rab5-Rab7 switch which is mediated by the complex Mon1-Ccz1. This complex interacts with Vps18 and Vps11 to recruit and activate Rab7A on early endosomes, which allows the conversion to late endosomes. As it is known, Vps11 interacts with SARS-CoV-2 protein Orf3A, which may modify it as to avoids the Vps11-Rab7A interaction and so impeding the “cleaning” mechanism of the last on α-syn in brain cells. In a recent study, Miao et al. [85] have found that Orf3a does not interact with any of the autophagy proteins. However, they proved that autophagy is inhibited in SARS-CoV-2 infected cells. Their findings indicate that such inhibition occurs through the interaction of Orf3a with the HOPS complex, consisting of Vps11, Vps16, Vps18, and Vps33A, and the HOPS-specific subunits Vps39 and Vps41. In particular, they reported the sequestration of Vps39 by Orf3a causing accumulation of the HOPS protein on late endosomes/lysosomes. Both proteins, Vps11 and Vps39, which are found here as perturbators of PD VPs, may result in the inhibition of autophagy, thus resulting in PD. Other potential mechanism also exist, such as via the inhibition of the biogenesis of the phagophore, which depends on the functionality of Rab1a (see Reference [83] and refs. therein). We should remark here that macroautophagy is cross-linked with exosome biogenesis [86]. For instance, when autophagy or lysosomal misfunction prevent degradation of protein aggregates there is an increase in exosome release to alleviate proteoxomic stress [86]. This explain our mechanism of cascade from viral proteins to CNS effects via exosome-mediated transport of perturbators. It is worth mentioning here that the autophagy-exosome crosstalk has been found to play a role in neurodegenerative diseases like PD.

**Figure 4:**
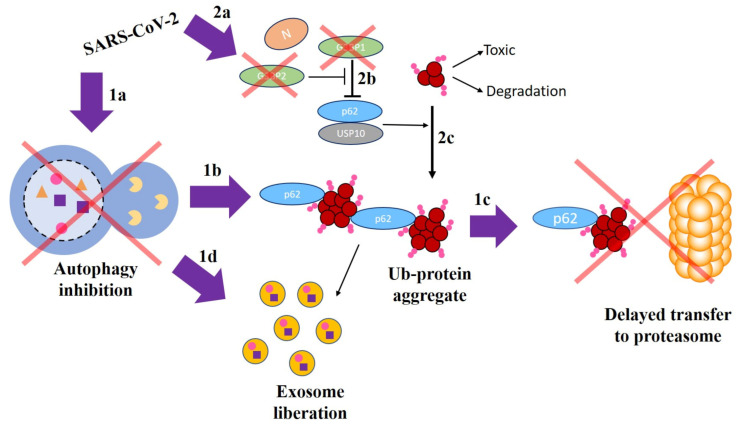
A schematic model explaining the involvement of NUP62 and G3BP1 proteins in the development of PD due to the effects of SARS-CoV-2 (see text for explanation).

Let us now return to the example provided in Figure 1 which involved the interaction of the viral nucleocapsid N protein with host G3BP1 and the interaction of this with NUP62. We will now explain this mechanism at the molecular level with the help of Figure 4. First, we should remark that SARS-CoV-2, similarly to other virus, inhibits autophagy in human host cells [85,87,88,89]. This is schematically represented in Figure 41a. It has been shown by Hyun et al. [90] that this inhibition causes accumulation of NUP62 and protein aggregates (Figure 41b), with a subsequent delayed migration of ubiquitinated substrates to the proteasomes (Figure 41c). NUP62 is a 520-amino acids protein associated with the nuclear envelope, which is found here to be perturbed by NUP88, NUTF2, RAE1, and G3BP1. We should notice that the interaction NUP62-G3BP1 was not among those reported in the database STRING (see Methods), but it is reported in a recent work by Anasimov et al. in 2019 [91]. These four perturbators interact with SARS-CoV-2 proteins Nsp9, Nsp15, Orf6, and N, respectively [65]. The protein NUP62 is involved in the formation of autophagosomes and its defective function or altered modulation is known to be associated with the pathogenesis of PD [90,91,92,93,94]. Indeed, NUP62 is known to induce protein aggregation and aggresome formation, which, in the case of aggregate formation of α-syn, produces PD.

On the other hand, Nabeel-Shah et al. [95] have reported that SARS-CoV-2 N protein sequesters G3BP1 and G3BP2 through its strong physical interaction with these proteins, which attenuate stress granule (SG) formation (Figure 42a). It is known that N protein is a multifunctional protein which is involved in many aspects of viral life cycle [96,97,98]. Anisonov et al. [91] have found that G3BP1 (Ras GTPase-activating protein binding site 1) inhibits ubiquitinated protein aggregations induced by NUP62 (Figure 42b). That is, G3BP1 is a negative regulator of protein aggregation, such that depletion of G3BP1 stimulates NUP62-induced aggregation of α-syn and aggresome formation (Figure 42c). It is important to remark here that ubiquitination plays a role, together with the inibition of the autophagy, in the selective incorporation of exosomal proteins, which may explain the formation and excessive liberation of exosomes containing perturbator cargoes as hypothesized in this work (Figure 41d). The mechanism is completed by the G3BP2 attenuation of G3BP1 inhibitory effect by competing with the G3BP1-NUP62 interaction (Figure 42b). Then, because N protein targets G3BP1 and G3BP2 for SG formation, by means of strong PPI, the ubiquitinated oligomers are not complexed into aggregates (formed anyway due to autophagy inhibition). These ubiquitinated oligomers are known to be toxic to the cells [91].

Therefore, both pathways may end up in PD. The inhibition of autophagy by the formation of ubiquitinated protein aggregates and the excessive formation of exosomes which may contain perturbators, and the sequestration of G3BP1 and G3BP2 by the viral N protein may also triggers NUP62-induced aggregation of α-syn. It should be remarked that G3BP1 is also a perturbator for the PD-VPs USP10 [91], GiGYF2 [99], EIF4G1 [100], and TIAL1 [101]. The first protein (USP10), which promotes protein aggregation and aggresome formation, interacts with NUP62 increasing NUP62-induced protein aggregation [91]. Anisonov et al. [91] also found that USP10 is inhibited by G3BP1 in a mechanism similar to that for NUP62, which indicates an analogous cascading route from SARS-CoV-2 to PD for this protein. For the other proteins, it is possible that other mechanisms are involved. For instance, a mutation situated in the GYF domain of GIGYF2 which disrupt the ligand-binding abilities of this domain, has been found to be associated with PD [99]. In the case of EIF4G1, it was linked to PD in a recent study after some conflicting results on its role in familial Parkinsonism [100]. Finally, TIAL1 has been found with significant alterations in motor cortex of postmortem brain donors with PD [101].

The most abundant PTM that viral proteins can induce in host proteins is their phosphorylation. Bouhaddou et al. [66] have reported that 40 out of the 332 host proteins interacting with SARS-CoV-2 are significantly differentially phosphorylated upon infection by SARS-CoV-2 (for statistical criteria see Bouhaddou et al. [66]). Among these 40 significantly differentially phosphorylated host proteins, there are 6 identified here as perturbators of PD-VPs. They are: MARK2, HDAC2, LARP7, PRKACA, PRKAR2A, and PRKAR2B. In total, these proteins are found to perturb 10 PD-VPs, namely: BRAF, EZH2, FOX01, LIN28A, MAP2, MAPT, MTA1, PRKACA, PRKACB, and PRKAR1B. From these genes, PRKACB is perturbed by three proteins: PRKACA, PRKAR2A, and PRKAR2B, which interact with the viral proteins Nsp13, Nsp14, and Nsp15. PRKACB is a member of the serine/threonine protein kinase family known to mediate signaling via cyclic adenosine monophosphate, which has been found to be downregulated in patients with PD [102]. Therefore, it is plausible that the differential phosphorylation of the perturbators of this protein, PRKACA, PRKAR2A, and PRKAR2B, produces its downregulation and may trigger Parkinsonism. Notice that one of these perturbators, PRKACA, which is significantly expressed in both the lungs and the CNS, has also been reported as potentially implicated in PD by modulating MAPK and insulin signaling pathways [103]. It is perturbed by two perturbators: PRKAR2A, PRKAR2B. The rest of the PD-VPs mentioned before are perturbed by only one perturbator. Several of these PD-VPs, apart from PRKACB, have been reported to be downregulated or having lost their functions in PD. These are the cases of FOXO1 [104], LIN28A [105], and MAP2 [106]. Thus, it is plausible that they are affected by the significant PTM of their perturbators, as in the case of PRKACB. The protein EZH2 [107] has been found to be involved in proteosomal degradation of α-syn and alteration on its levels has been implicated in PD, possibly due to similar reasons as for the case of NUP62. The implication of MAPT [108,109,110], which is known as tau protein, is well known for its implication in Alzheimer disease, but it has also been found associated with PD. MTA1 is an upstream modulator of tyrosine hydroxylase and has been found to play a significant role in PD pathogenesis [111]. Finally, BRAF is known to play a role in neuronal survival and maturation. Its connection with PD has been linked through its interaction with RIT2, which is a PD risk factor in Asian and Caucasian cohorts [112]. The remaining 27 PD-VPs are perturbed by only one perturbator each.

Finally, we would like briefly to consider the role played by some of the most important perturbators found here. There are three perturbators, G3BP1, OS9, and SCCPDH, which perturb 5 PD-VPs each. We have previously considered G3BP1, so that we focus now on OS9 and SCCPDH. Osteosarcome 9 (OS9) is a component of the endoplasmic reticulum (ER)-associated degradation (ERAD) machinery [113]. The main function of ERAD is to retrotranslocate to the cytoplasm the unfolded or malfolded proteins which are retained in the ER, such that they can be degraded by the proteosome. In particular, OS9 is responsible for the recognition of unfolded proteins to which it binds to. Failing such recognition will leave unfolded/malfolded proteins in the ER which may expose their hydrophobic amino acids, increasing their tendency to form protein aggregates. Therefore, there are many reports linking ER stress and neurodegenerative diseases, such as PD (see Reference [113] and refs. therein). OS9 forms a complex with SARS-CoV-2 Orf8 protein [65]. This somehow enigmatic 121-amino acids protein has a relatively high number of disulfide bridges, which points out to its residence in the ER [114]. Thus, this joint habitability of Orf8 and OS9 in the ER may increase the chances of modification of the host protein impeding the realization of its important function. This PTM of OS9 may also modify its interaction with AMFR [115], P4HB [116], SEL1L [117], SYVN1 [117], and VCP [118], which are found here as PD-VPs. For instance, SEL1L [117] has been suggested to be the ERAD tuning receptor, which selectively captures OS9–by means of a PPI–for constitutive clearance from the ER. Thus, there are many routes linking OS9 disruption by SARS-CoV-2 with PD. The other major perturbator of PD-VPs, SCCPDH, is targeted by viral Nsp7 [65]. There is not much information about the possible role that this host protein could have in PD. However, we have found that 3 of the five PD-VPs that interact with it appear upregulated/significantly-increased in blood (APP [119] and SERPINE1 [120]) or CSF (TGFB1 [121]) of PD patients. The other two PD-VPs are neuroprotective for PD (HGF [122] and TIMP1 [123]). Therefore, we may suggest two potential alternative mechanisms involving the interactions of SCCPDH with PD-VPs. The two perturbators interacting with 4 PD-VPs each, PRKACA and VPS39, were already analyzed here. Other possible mechanistic links can be extracted from the rest of perturbators and their PD-VPs, which, in general, can serve for proposing and validating research hypothesis related to the potential impact of COVID-19 on CNS, in general, and with Parkinsonism, in particular.

## 6. Conclusions

Here, we provide a mechanism explaining how the damages produced by the interactions of SARS-CoV-2 proteins with human proteins expressed in the lungs can cascade to affect proteins mainly expressed in the CNS, which trigger the development of PD. This mechanism is based on the modern paradigm stating that diseases are mainly produced by the disruption of PPI networks. In this case, we consider that some proteins expressed significantly in the lungs can be post-translationally modified by SARS-CoV-2 proteins. These proteins can act as perturbators of PD-VPs if they can be encapsulated in exosomes which then navigate up to the CSN. We have identified 24 perturbators, damaged in the lungs by SARS-CoV-2 proteins, which may disrupt the normal functioning of 44 PD-VPs triggering Parkinsonism. Several mechanisms are devised here for the cascading of effects from the lungs to PD. We have found that interfering with autophagy/ubiquitination processes triggers the generation of large amounts of exosomes containing perturbators in their cargoes, which would insult several PD-VPs. These findings offer a great opportunity for testing several hypothesis about the potential CNS damage of SARS-CoV-2 and possibly other virus, as well as to confirm some mechanisms involving new potential biomarkers for PD.

## Figures and Tables

**Table 2:** Proteins found here to be perturbators of PD-VPs. Biological functions are reproduced from Gordon et al. [65] and the data about protein and RNA expressions are taken from The Human Protein Atlas [77] (https://www.proteinatlas.org/ accessed on 15 December 2020). See Methods for details.

Protein	Biological Function	Protein Expression	RNA Expression (pTPM)
BRD4	-	high	29.6
CEP350	centrosome	high	11.5
CSNK2A2	stress granules	-	40.9
ECSIT	respiratory electron transport	medium	30.8
G3BP1	stress granules	medium	41.0
HDAC2	-	high	44.6
ITGB1	-	-	317.6
LARP7	7SK snRNP	-	49.0
MARK2	MARK kinases	-	18.5
NUP88	nuclear pore	medium	11.1
NUTF2	-	low	62.0
OS9	ER protein quality control	high	179.9
PRKACA	protein kinase A signaling	medium	68.5
PRKAR2A	protein kinase A signaling	high	13.0
PRKAR2B	protein kinase A signaling	low	11.2
RAB1A	Rab signaling	low	167.3
RAB14	Rab signaling	medium	55.6
RAE1	nuclear pore	-	15.3
RHOA	-	medium	554.4
RTN4	ER morphology	-	300.8
SCCPDH	-	low	20.3
VPS11	HOPS complex	medium	-
VPS39	HOPS complex	-	30.6

## Data Availability

The datasets used and/or analyzed in the current study are available from the author on reasonable request.

## Abbreviations

AMFR: Autocrine motility factor receptor; APP: Amyloid beta precursor protein; BRAF: B-Raf proto-oncogene, serine/threonine kinase; BRD4: Bromodomain-containing protein 4; cAMP: Cyclic adenosine monophosphate; Ccz1: Calcium caffeine Zinc sensitivity; CD47: Cluster of differentiation 47; CEP350: Centrosome-associated protein 350; CNS: Central nervous system; COVID-19: Coronavirus disease 2019; CSF: Cerebro spinal fluid; CSNK2A2: Casein kinase II subunit Alpha; ECSIT: Evolutionarily conserved signaling intermediate in Toll pathway; EIF4G1: Eukaryotic translation initiation factor 4 gamma 1; ER: Endoplasmic reticulum; ERAD: ER-associated degradation EZH2:Enhancer of zeste homolog 2; FOX01: Forkhead box protein O1; G3BP1: Ras GTPase-activating protein-binding protein 1; G3BP2: Ras GTPase-activating protein-binding protein 2; GDA: Genedisease associations; GIGYF2: GRB10 interacting GYF protein 2; GTEx: Genotype-Tissue Expression; GTPase: Guanosine triphosphate hydrolase; GYF: Glycine-tyrosine-phenylalanine; HDAC2: Histone deacetylase 2; HGF: Hepatocyte growth factor; HOPS: Homotypic fusion and protein sorting; IgG: Immunoglobulin G; ITGB1: Integrin subunit beta 1; LARP7: La Ribonucleoprotein 7; LIN28A: Lin-28 homolog A; MAP2: Microtubule-associated protein 2; MARK: Microtubule affinity regulating kinase; MARK2: Microtubule affinity regulating kinase 2; Mon1: Monensin sensitivity 1; MRI: Magnetic resonance imaging; MTA1: Metastasis-associated protein 1; N: Nucleocapsid; NSP13: Nonstructural protein 13; NSP14: Nonstructural protein 14; NSP15: Nonstructural protein 15; NSP9: Nonstructural protein 9; NUP62: Nucleoporin 62; NUP88: Nucleoporin 88; NUTF2: Nuclear transport factor 2; Orf3A: Open reading frame 3A; Orf6: Open reading frame 6; Orf8: Open Reading frame 8; OS9: Osteosarcoma amplified 9; P: Perturbator; P4HB: Beta-subunit of prolyl 4-hydroxylase; PD: Parkinson’s disease; PPI: Protein-protein interaction; PRKACA: Protein kinase cAMP-activated catalytic subunit Alpha; PRKAR1A: Protein kinase cAMP-dependent type I regulatory subunit Alpha; PRKAR2A: cAMP-dependent protein kinase type II-alpha; PRKAR2B: cAMP-dependent protein kinase type II-beta; PTM: Post-translational modification; pTPM: Protein-coding transcripts per million; RAB: Ras-related protein Rab; RAB14: Ras-related protein Rab-14; RAB1A: Ras-related protein Rab-1A; Rab1B: Ras-related protein Rab-1B; RAB5: Ras-related protein Rab-5; Rab6B: Ras-related protein Rab-6B; RAB7A: Ras-related protein Rab-7A; RAB8A: Ras-related protein Rab-8A; RAE1: Ribonucleic acid export 1; Ras: Rat sarcome; RHOA: Ras Homolog Family Member A; RING: Really interesting new gene; RIT2: Ras-like protein expressed in neurons; RNA: Ribonucleic acic; RTN4: Reticulon 4; RTN4R: Reticulon 4 receptor; RT-PCR: Real-time polymerase chain reaction; RUNX1T1: RUNX1 partner transcriptional co-repressor 1; SARS-CoV-2: Severe Acute Respiratory Syndrome coronavirus 2; SCCPDH: Saccharopine Dehydrogenase (Putative); SEL1L: Protein sel-1 homolog 1; SERPINE1: Serpin family E member 1; SG: Stress granule; SLC2A4: Solute carrier family 2 member 4; snRPN: small nuclear ribonucleoprotein SYVN1: Synoviolin 1; TGFB1: Transforming growth factor-beta 1; TIAL1: Cytotoxic granule associated RNA binding protein like 1; TIMP: tissue inhibitor of metalloproteinases; TIMP1: TIMP metallopeptidase inhibitor 1; Ub: ubiquitinated; USP10: Ubiquitin specific peptidase 10; VP: Vulnerable protein; VPC: Valosin-containing protein; VPS11: Vacuolar protein sorting-associated protein 11; VPS16: Vacuolar protein sorting-associated protein 16; VPS18: Vacuolar protein sorting-associated protein 18; VPS33A: Vacuolar protein sorting-associated protein 33A; VPS39: Vacuolar protein sorting-associated protein 39; VPS41: Vacuolar protein sorting-associated protein 41; *α*-sync: *α*-syncuclein.
